# Development and Validation of Manually Modified and Supervised Machine Learning Clinical Assessment Algorithms for Malaria in Nigerian Children

**DOI:** 10.3389/frai.2021.554017

**Published:** 2022-02-03

**Authors:** Megan McLaughlin, Karell G. Pellé, Samuel V. Scarpino, Aisha Giwa, Ezra Mount-Finette, Nada Haidar, Fatima Adamu, Nirmal Ravi, Adam Thompson, Barry Heath, Sabine Dittrich, Barry Finette

**Affiliations:** ^1^ THINKMD, Burlington, VT, United States; ^2^ FIND, Geneva, Switzerland; ^3^ Network Science Institute, Northeastern University, Boston, MA, United States; ^4^ Santa Fe Institute, Santa Fe, NM, United States; ^5^ Vermont Complex Systems Center, University of Vermont, Burlington, VT, United States; ^6^ eHealth Africa, Kano, Nigeria; ^7^ Department of Pediatrics, University of Vermont, Burlington, VT, United States

**Keywords:** digital health, IMCI, rapid diagnostic test, machine learning, febrile illness

## Abstract

It is currently estimated that 67% of malaria deaths occur in children under-five years (WHO, 2020). To improve the identification of children at clinical risk for malaria, the WHO developed community (iCCM) and clinic-based (IMCI) protocols for frontline health workers using paper-based forms or digital mobile health (mHealth) platforms. To investigate improving the accuracy of these point-of-care clinical risk assessment protocols for malaria in febrile children, we embedded a malaria rapid diagnostic test (mRDT) workflow into THINKMD’s (IMCI) mHealth clinical risk assessment platform. This allowed us to perform a comparative analysis of THINKMD-generated malaria risk assessments with mRDT truth data to guide modification of THINKMD algorithms, as well as develop new supervised machine learning (ML) malaria risk algorithms. We utilized paired clinical data and malaria risk assessments acquired from over 555 children presenting to five health clinics in Kano, Nigeria to train ML algorithms to identify malaria cases using symptom and location data, as well as confirmatory mRDT results. Supervised ML random forest algorithms were generated using 80% of our field-based data as the ML training set and 20% to test our new ML logic. New ML-based malaria algorithms showed an increased sensitivity and specificity of 60 and 79%, and PPV and NPV of 76 and 65%, respectively over THINKD initial IMCI-based algorithms. These results demonstrate that combining mRDT “truth” data with digital mHealth platform clinical assessments and clinical data can improve identification of children with malaria/non-malaria attributable febrile illnesses.

## Introduction

Globally, it is currently estimated that every two minutes a child under the age of five years dies from malaria. In Nigeria, where the prevalence of malaria is extremely high, malaria accounts for 9.8% of all deaths of children under-five and has prevalence close to 50% in the South West, North Central, and North West regions of Nigeria (Observatory, 2017; Organization, 2019; UNICEF, 2019).

A multiple indicator cluster Nigeria survey revealed that only 63.4% of children with a history of fever sought care from a health facility or provider. Of those who presented for care, only 13.8% received a malaria diagnostic test, with only 36.8% receiving antimalarial treatment, 20.6% being artemisinin-based combinations therapies (ACTs). In the Kano State region it was observed that 25% percent of children with fever sought care from a public facility, 14% from private facility and 1.9% from community health workers. Only 11.3% of these children received a malaria blood test. For children testing positive for malaria, only 9.1% were given ACT treatment, while 29.4% were given an antibiotic (Fund, 2017). These findings indicate that to improve mortality and morbidity of febrile children with malaria, there needs to be a significant increase in quality clinical risk assessment screening of children with fever linked with diagnostic testing and improved appropriate therapeutic intervention for children with positive malaria diagnostic tests.

Over 20 years ago, the WHO and UNICEF introduced community (iCCM) and clinic-based (IMCI) protocols for frontline health workers (FHWs), currently being used in paper-based forms or digital mobile health (mHealth) platforms, to reduce child mortality and morbidity in developing countries and in particular for malaria (Gera et al., 2016). Unfortunately in Nigeria, less than 25% of first level facilities, and 60% of health workers who care for sick children were trained in IMCI protocols since being introduced in 1997 (Fund, 2017). Identifying febrile children who are at specific risk for malaria is very challenging, especially in resource poor countries. As an acute febrile illness, malaria presents similarly to most other febrile illnesses (fever, headache, vomiting, respiratory symptoms, and cold chills, etc.). A study investigating severe malaria indicated identification of malaria risk using only IMCI protocol was correct less than 30% of the time (Unitaid, 2018). This led to the WHO recommending that all suspected cases of malaria, identified using IMCI protocol, have a parasitological confirmation [blood smear, DNA amplification, and/or malaria rapid diagnostic test (mRDTs)] before treatment, to promote rational use of ACTs and antimicrobials.

Malaria rapid diagnostic tests (mRDT) are a simple and cost-efficient method for screening for malaria by FHWs (Mokuolu et al., 2016). A limitation of the current IMCI/ICCM algorithms is the limited access to alternative treatment and diagnostic tools beyond the mRDT. This contributes to poor linkage of clinical risk assessments, adherence to mRDT administration, inappropriate use of ACTs, as well as inappropriate prescription of antibiotics. Health workers also expressed uncertainty about how to manage non-malaria fevers because of a fear of making the wrong decision and losing patients to follow-up, that could ultimately lead to death, which would further decrease caregiver’s trust in their effectiveness (Johansson et al., 2016).

As such, there is a significant need for improved approaches for assessing and treating children with acute febrile illnesses that provide a more standardized approach leading to increased certainty about the causes of fever and the proper treatment and follow-up of patients. The ability to modify digital mHealth platform algorithms based on data driven analysis and testing, as well as using such data with diagnostic field-based diagnostic data to develop supervised Machine Learning (ML) algorithms, could prove to be a valuable approach to improve the accuracy of malaria assessment in children with malaria by FHWs.

Current ML studies have demonstrated their utility in identifying risk for various diseases including diabetes, tuberculosis, and cancer (Shinde and Rajeswari, 2018). To date, there is no evidence demonstrating the utility of ML-based malaria assessment algorithms as part of a digital mHealth tool leading to improved malaria risk assessments by FHWs. Using ML to accurately identify risk of specific illnesses based on current and historical datasets can be invaluable for the creation of specific clinical assessment algorithms for various febrile illnesses such as malaria (Obermeyer and Emanuel, 2016; Rajkomar et al., 2018).

In this study, we demonstrate the development and testing of new supervised ML malaria-risk algorithms, using field-based malaria-risk assessments, clinical data, and mRDT diagnostic data generated via the use of THINKMD’s IMCI compliant mHealth platform, that could improve the identification of malaria in children with febrile illnesses by FHWs.

## Materials and Methods

### Study Design

From July to August 2018, THINKMD’s application was utilized by seven Community Health Workers (CHWs) for four weeks in five primary health care facilities in Local Government Areas (LGA) of Kano State, Nigeria.

**FIGURE 1:**
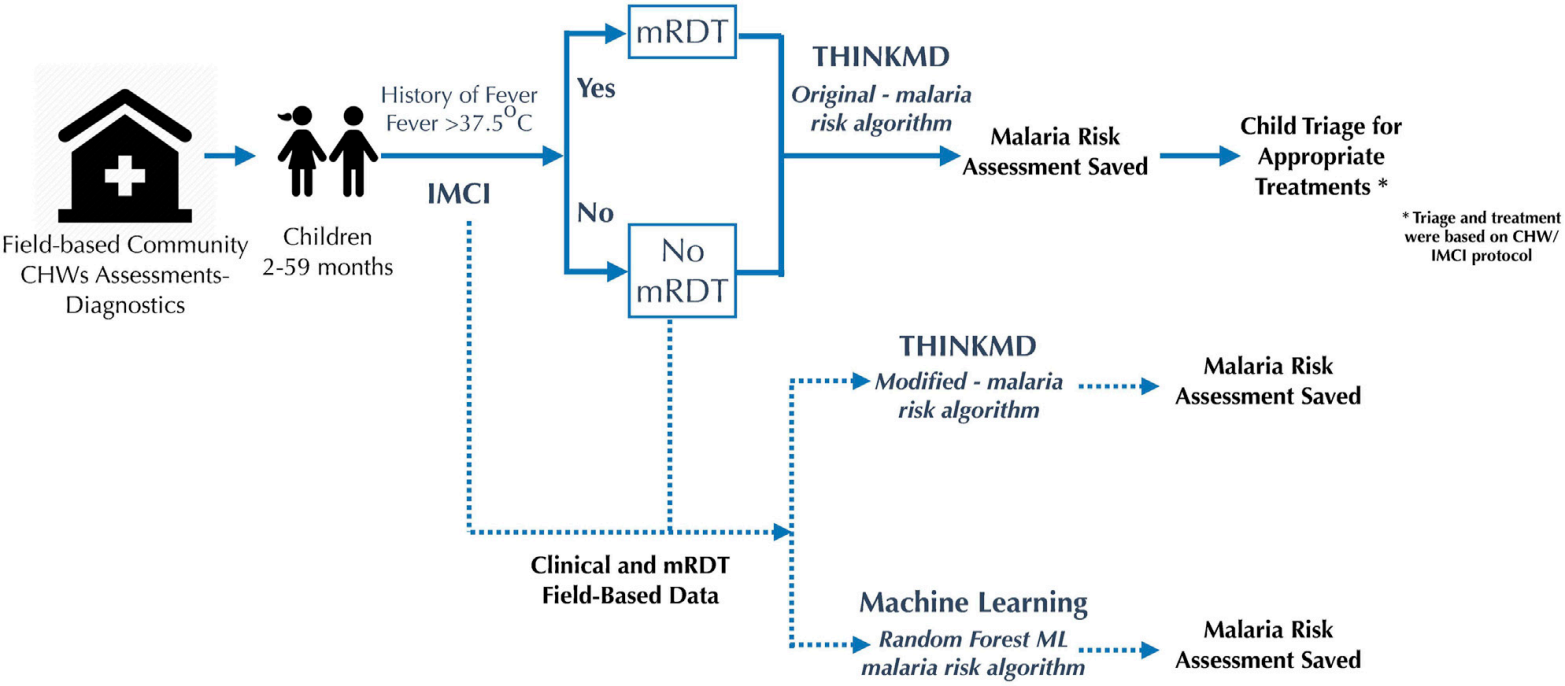
Patient flow.

Assessments using the THINKMD mHealth tool with embedded malaria rapid diagnostic test (mRDT) were performed by CHWs on all children 2–59 months who presented with fever or history of fever over a four-week period to five participating clinics. Integrated Management of Childhood Illness (IMCI) designation of malaria risk for children were based on Nigeria’s standard IMCI protocol for febrile illness assessment, specifically any child presenting with a history of fever OR having a current fever (>37.5°C) based on a thermometer-based measurement would be determined to be at risk for malaria and receive an mRDT. As such, recommendations of mRDT testing had the lowest possible IMCI recommended threshold. The data collected during the study were compared with historic data collected from the last 12 months from the DHIS2 Kano State region dataset provided by the Ministry of Health. For children not presenting with history of fever or with an axillary temperature, the mRDT test was not recommended. As a result, there were no individuals included in the data set deemed to not be at risk due to those criteria. All children were referred and/or treated as recommended by WHO and IMCI guidelines. Figure 1, Study Design.

### Technology

For this study we utilized THINKMD’s IMCI compliant mHealth platform (Finette et al., 2019), with embedded mRDT workflow and result capture capability. This tool obligates FHW users to acquire over 94% of all recommended IMCI data points to generate integrated clinical risk assessments for the most important clinical conditions affecting children from 2 months to 5 years, including malaria (Finette et al., 2019). Previous validation results demonstrate that clinical assessments completed by FHW using THINKMD’s mHealth tool had a specificity correlation between 84 and 99% to local health professionals performing blind assessments of the same child (Finette et al., 2019). In addition, results from a separate study have demonstrated that utilization of the THINKMD mHealth tool resulted in a mean increase in WHO-IMCI adherence of 41% (from 30.6 to 71.4%) when observed, a WHO-IMCI adherence mean increase of 50.4% (from 30.6 to 81.0%) when captured through the THINKMD mHealth platform, as well as increased acquisition of IMCI key danger signs by >40%. This paper is currently in preparation.

### Embedded Malaria Rapid Diagnostic Test Workflow for Community Health Workers

Nigeria’s standard IMCI workflow for febrile illness assessment specifies that any child presenting with a history of fever *or* having a current fever based on a thermometer measurement is at risk of malaria and should receive an mRDT, as mentioned above. In accordance with this policy, the THINKMD platform was modified to include a diagnostic step in the fever management branch whereby children presenting with a history of fever or a recorded axillary temperature of >37.5°C were recommended an mRDT. For children not presenting with history of fever or with an axillary temperature <37.5°C the mRDT test was not recommended.

**FIGURE 2:**
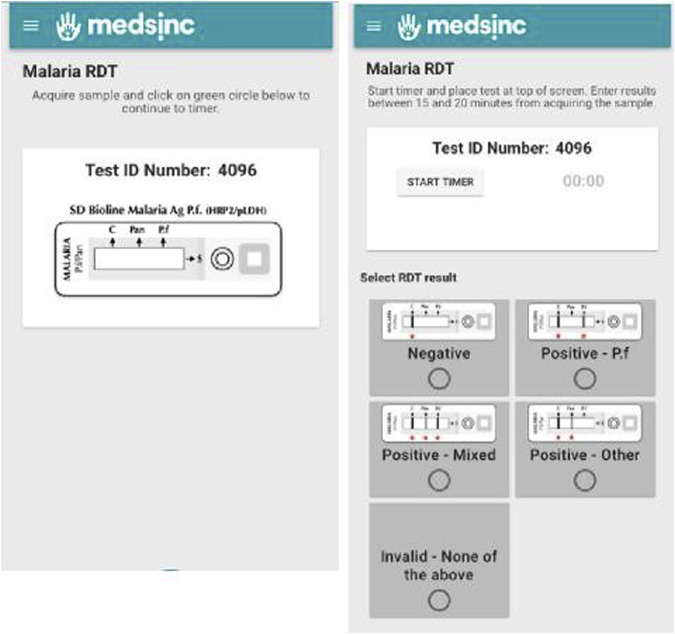
Embedded mRDT workflow panels for acquiring and reporting mRDT results by CHWs.

Workflow for acquiring and documentation of mRDT results were embedded into the THINKMD mHealth tool to standardize and ensure consistency of mRDT testing and results acquisition by CHWs. mRDT workflow panels included an automatically generated lab ID number, an embedded timer set appropriately for the specific mRDT test, and a panel for test results input. Figure 2. To improve accuracy of mRDT results, CHWs were only able to record mRDT results within the 15–20-min manufacturer recommended time frame. If this time window was missed, they were required to repeat the mRDT.

### Study Sites and Participants

This study was conducted for 4 weeks in 5 primary health care facilities in Local Government Areas (LGA) of Kano State, Nigeria. These included 2 health clinics (Alfindinki and Tudun Murtala) and 3 health posts (Kantudu, Unguwa Jakada and Hotoro Danmarke). Study participants were children aged 2–59 months presenting with fever or a history of fever at the health care facilities.

Seven CHWs were trained for 1 day to use the THINKMD mHealth platform with the additional malaria diagnostic step. Nigerian CHWs in Kano State already conduct mRDTs in their regular routine as per Nigerian IMCI guidelines, therefore training emphasized following the manufacturer’s instructions on test processing, and time window to read and input results.

This study was approved by the Committee on Human Research in the Medical Sciences at the University of Vermont Center, Vermont, United States, as well the Ministry of Health, Kano State, Nigeria.

### Statistical Methods

THINKMD utilized two independent approaches to clinical logic refinement and testing: one that focused on modifications of its current “physician based” core logic by changing logic parameters of specific clinical data elements and the tolerance scores used to determine clinical risk for malaria; the other being the development of ML logic initially developed from our current logic algorithms and testing using field-based truth data.

The main goals of our analysis were to:

• Refine THINKMD mHealth malaria “physician assessment” logic based on field-based data acquired in this study and compare the new logic assessments using the field-based datasets.
• Improve malaria risk assessment logic to improve identification of acute febrile patients who should receive mRDT screening and reduce the number where it is not indicated.
• Develop ML based malaria assessment logic using a supervised ML approach using field-based THINKMD malaria assessment data and algorithms, as well as current field-based data elements and mRDT truth data.

For this study, we initially tested THINKMD malaria risk assessment algorithms for field-based screening that had a higher criterion for generating a risk assessment for malaria than the IMCI based guidelines, the latter requiring that children had history or actual fever at presentation. This THINKMD malaria algorithm set was called, “original malaria algorithms.” These original malaria algorithms required children to have evidence of fever, as above, as well as some evidence of a systemic infection/sepsis utilizing additional specific malaria presenting symptoms, vital signs, and physical findings.

Specifically:

Fever + evidence of sepsis + 1 malaria specific symptom = Risk of Malaria

To test whether we could modify these algorithms and test them using the same field-based clinical encounter data, we decreased the stringency of our assessment logic to more align with IMCI malaria risk assessment. These algorithms were called “modified malaria algorithms” which includes fever as a mandatory criterion as well as 1 additional key malaria symptom/clinical finding, such as headache or vomiting, for example.

Fever + 1 malaria specific symptom = Risk of Malaria

Malaria Specific Symptoms:

• Fever
• Aching
• Cold chills
• Headache
• Sepsis
• Pain when moving neck
• Pale eyelids
• Vomiting

Our interest in testing more stringent original malaria assessment logic was to gain insights whether we could accurately identify who presented with fever from a non-malaria etiology, and to identify malaria more accurately in children with history of fever. We then utilized the field-based encounter data acquired in this study to manually test the accuracy of the alternative less stringent modified malaria algorithms.

Using paired mRDT results with clinical health data and THINKMD generated malaria risk assessments, we were able to perform a correlative analysis of the accuracy for our original and modified malaria risk algorithms compared to Nigeria’s paper-based adaptation of standard IMCI identification of malaria risk. Comparative analysis of mRDT findings provided insights into the accuracy of both THINKMD malaria risk assessment algorithms with the goal of distinguishing patient’s presenting with febrile illness symptoms to ensure appropriate diagnostic testing and therapeutic intervention.

We initially performed a comparative statistical analysis between THINKMD malaria risk assessment to Nigeria’s IMCI paper-based approach and mRDT results using historical DHIS2 data collected within Local Government Area clinics in Kano State and reported monthly to the regional/national DHIS2 system throughout the year 2017. We then compared these data sets with the original malaria algorithms, as well as with an alternative modified malaria algorithm, comparing each to historical and current IMCI malaria-risk assessments.

To generate ML based algorithms using the mRDT results, we used random forest models to predict mRDT results using clinical data captured in THINKMD’s mHealth platform as implemented in the R package random Forest v4.6-14 (Liaw and Wiener, 2002). Briefly, we trained an ensemble of classification trees by generating 1,000 bootstrapped samples from our data with size equal to the total number of patients. This was done using the clinical data sets acquired by using the THINKMD risk assessment algorithms and the acquired mRDT results. Then, at each node in the tree, we randomly selected 50 predictors and chose the best subset of predictors to keep at each node using binary recursive splitting evaluated with a Gini index over our two classes (positive vs. negative mRDT result) (Breiman, 2001). The resulting trees are not pruned. To classify patients, i.e., predict the mRDT result, patient data are evaluated by each of the 1,000 resulting trees. The most estimated class over all the trees, i.e., mRDT result with the most vote, is the final prediction. Variable importance was assessed based on the percentage of time each case in the out-of-sample data set was misclassified when a given variable was excluded from the predictor set. For more detailed information, see Breiman (2001).

Random forest ML-based development is considered a supervised ML approach compared to other methodologies and provides several advantages to other ML approaches in that the path to the endpoint ML is known, and it allows for the analysis of each data point and its importance in the generated ML logic. This can ultimately allow for platform modifications, importantly, the prioritization of data point acquisition to get to the ultimate appropriate assessment, thus streamlining platform design and data acquisition while maintaining accuracy. For ML accuracy confirmation, the new clinical ML-based random forest algorithms can then be tested against a specific set of field-based testing sets that were not used to train the ML decision trees. The ML algorithm had access to the same information as the THINKMD risk assessment. The specific variables available for the machine learning algorithm are listed in Supplementary Table S1.

To assess the generalizability of our results, we performed n-fold cross validation by iteratively holding out 10% of the data, fitting the random forest on the remaining 90% and evaluating the results on the held-out set and performed leave-one-out cross validation by iteratively holding out all data from each of the five clinics, training on data from the remaining four and evaluating on the held-out set. Models were compared using sensitivity, specificity, positive predictive value (PPV), negative predictive value (NPV), and the F1 score (a composite of precision and recall).

To evaluate how many patient encounters are needed to effectively “train” the random forest model, data were incremented by subsampling 5% of the total data set in each simulation (5, 10, 20 ... 90%). For each subsample size, 1,000 training and testing (90/10 splits) loops were generated.

### Malaria Rapid Diagnostic Test

SD Bioline Malaria Ag Pf/Pan RDTs (Standard Diagnostics Inc., Republic of South Korea) were provided to all study sites throughout the duration of the study and performed based on manufacturer’s instructions and guided by the app. This mRDT detects HRP-2 antigen specific to *Plasmodium falciparum* and pLDH exhibited by all Plasmodium species.

### Data Safety and Storage

Data were protected and stored on International Business Machines Cloudant Database as a Service. All access to cloud and data based is encrypted *via* HTTPS and passed through a REST full application programming interface with full authentication. There were nightly backups and replication of databases.

Datasets are available on request. Due to the sensitivity of the information collected, partners require all appropriate approvals by participating parties prior to sharing of data for any reason.

## Results

A total of 555 children were assessed by seven CHW at five sites during this one-month study. Among 480 children identified as at risk of malaria following IMCI guidelines, 66.7% had positive mRDT results and 33.3% had negative mRDT results. Table 1 summarizes the total assessments and RDT results for all children presenting to clinics for evaluations.

**TABLE 1:** Summary of clinical malaria assessments and malaria RDT (mRDT) results.

Total children seen during the study period	555
Total children with identified malaria risk as per IMCI	480
Total excluded based on no identified malaria risk as per IMCI	72
Total children with THINKMD positive malaria risk (original algorithm)	205
Total children with THINKMD negative malaria risk (original algorithm)	350
Total mRDTs administered	480
Total negative mRDT tests	160
Total positive mRDT for malaria (combination of the below)	320
Total positive for mRDT for *Plasmodium falciparum* (Pf) malaria	280
Total positive mRDT for mixed malaria parasites	33
Total positive for other malaria parasites	7
Total timed-out mRDTs	3
Total inconclusive mRDTs	0

### Comparison Between Paper Based IMCI Malaria Guidelines to THINKMD’s Malaria Risk Assessment Algorithms

A comparison of positive predictive values (PPV) for malaria assessments was performed between historical IMCI malaria assessment data from DHIS2, Table 2, current IMCI data and for THINKMD original malaria algorithm, Table 3. When calculating the measures presented in Table 3, we used the mRDT result, i.e., positive or negative for malaria, as the ground truth. We expand on the potential implications of our decision to use the mRDT results as the comparator in the discussion.

**TABLE 2:** Historical DHIS2 data for malaria in Kano State, Nigeria 2017.

Total number of children with fever	4,621
Total number of mRDTs performed	4,595
Total number of (+) mRDTs	2,305 (True positives)
Total number of (−) mRDTs	2,290 (False positives)

**TABLE 3:** Dichotomized statistical analysis between historical and current IMCI and original THINKMD malaria risk assessments algorithms.

Statistical analysis	IMCI-historic	IMCI-current	Original THINKMD malaria algorithm
Sensitivity, (%)	NA	NA	43
Specificity, (%)	NA	NA	64
PPV, (%)	50.1	67	71
NPV, (%)	NA	NA	36

Abbreviations: IMCI, Integrated Management of Childhood Illness; mRDT, malaria rapid diagnostic test; PPV positive predictive value; NPV, negative predictive value.

One limitation of this analysis was that we were not able to determine Negative Predictive Values (NPV) for the IMCI data sets, as assessments identified as “no malaria risk” did not have an mRDT performed. Comparative analysis for PPV for malaria risk assessment between current and historic IMCI and original risk algorithms are summarized in Table 3.

Despite the PPV being similar between the current IMCI and original risk assessment algorithms the IMCI assessment captured more children with malaria than the original risk algorithm since the latter had more stringent criteria for generating a positive malaria risk assessment which is reflected with a low sensitivity. The observed improvement in the current IMCI assessment could be related to improved training and/or the variability in malaria risk assessment during the entire year, which is seen in the historic IMCI data. Thus, when IMCI screening for high risk is used during low malaria risk season, PPV will decrease.

Of clinical importance is that many children who present for an acute illness health evaluation are found to have more than one clinical condition requiring therapeutic intervention. To see if we observed this in our sample, we looked at the distribution of other key integrated THINKMD clinical assessments and their associations with mRDT results. Interestingly, sepsis, a clinical condition indicative of infection, directly associated with fever, and highly correlated with malaria, was presenting quite evenly across both negative and positive mRDT results, Table 4.

**TABLE 4:** Distribution of clinical conditions and severity variables identified by THINKMD’s malaria risk algorithm compared to mRDT findings.

Clinical condition/Severity	Positive mRDT (*n* = 320)	Negative mRDT (*n* = 160)	Not administered (*n* = 72)
**Sepsis**			
None/Mild	47%	52%	83%
Moderate	53%	48%	16%
Severe	—	—	—
**Dehydration**			
Mild	88%	91%	94%
Moderate	10%	7%	4%
Severe	1%	2%	1%
**Malnutrition**			
Mild	65%	73%	64%
Moderate	34%	26%	35%
Severe	1%	>1%	1% (inconclusive)^a^
**Respiratory Distress**			
Mild	86%	84%	87.5%
Moderate	14%	16%	12.5%
Severe	—	—	—

a No weight or MUAC acquired for these assessments.

Following this analysis, we wanted to evaluate our ability to manually modify THINKMD’s malaria logic to increase its sensitivity by decreasing the stringencies to generate a malaria risk assessment, specifically the modified malaria risk algorithms. This algorithm would then be tested using the original field-based dataset and compared to the newly generated malaria risk assessments with the acquired mRDT truth data. The main change to this modified algorithm was to eliminate the criterion for evidence of sepsis in the malaria assessment as described above. A summary of these findings is summarized in Table 5.

**TABLE 5:** Performance statistics of modified vs. original malaria risk assessment algorithm compared to mRDT result.

Statistical analysis	Original malaria risk assessment algorithm		Modified malaria risk assessment algorithm	
—	(−) mRDT *n* = 160	(+) mRDT *n* = 320	(−) mRDT *n* = 160	(+) mRDT *n* = 320
Sensitivity, %	0.36	0.70	0.47	0.69
CI	0.30–0.41	0.63–0.76	0.36–0.57	0.65–0.74
Specificity, %	0.70	0.36	0.69	0.47
CI	0.63–0.76	0.30–0.41	0.65–0.74	0.36–0.58
PPV, %	0.63	0.43	0.23	0.87
CI	0.55–0.70	0.38–0.49	0.17–0.30	0.83–0.90
NPV, %	0.43	0.63	0.87	0.23
CI	0.38–0.49	0.55–0.70	0.83–0.90	0.17–0.30

Abbreviations: CI, Confidence interval; mRDT, malaria rapid diagnostic test; PPV, positive predictive value; NPV, negative predictive value.

For this analysis, using the same inputted data elements and the associated mRDT results for each patient we observed a significant increase in PPV for (+) mRDT for the modified vs the original malaria risk algorithms; a significant increase in NPV for (−) mRDT for the modified vs the original malaria risk algorithms; and an expected increase in sensitivity and decrease in specificity in the modified vs. the original malaria risk assessment logic. This analysis shows that using this approach, we can make modifications in the THINKMD malaria risk assessment algorithms and perform a statistical comparison using the same clinical data set and confirmatory mRDT data. This an important new approach for logic development that did not require new sets of field-studies to initially test.

### Validation and Comparison of ML Generated Malaria Risk Assessment Algorithms With Manually Developed Malaria Risk Algorithms Using mRDT Field-Truth Data

Table 6 summarizes a comparative analysis of ML based algorithms using both training sets and separate raw ML data sets vs field-based mRDT data sets. A standard random forest approach was employed, using 80% of our field-based data as our ML training set and 20% raw data to test newly generated ML logic. We observed a 98–99% correlation of our ML algorithms with our training data sets from field based clinical data vs mRDT results and between 70–99% correlation between ML generated malaria risk algorithms with the raw clinical field-based data sets and mRDT results that were not used for ML training.

**TABLE 6:** Confusion matrix for THINKMD ML malaria assessment.

mRDT result	ML training set malaria assessment correlation (%)	ML raw test data set malaria assessment correlation (%)
(−) mRDT	0.98	0.74
(+) mRDT (Pf)	0.99	0.70
(+) mRDT (mixed)	0.99	0.95
(+) mRDT (other)	0.99	0.99

Abbreviations: ML, machine learning; mRDT, malaria rapid diagnostic test; Pf, *Plasmodium falciparum*.

A statistical comparison between THINKMD’s original malaria risk algorithms and the newly generated ML malaria risk algorithms vs. mRDTs is shown in Table 7. Performance for both THINKMD and the ML malaria risk algorithm was quantified separately on both the training (80%) and testing (20%) portions of the data set.

**TABLE 7:** Performance statistics of THINKMD’s original malaria risk algorithms and the ML malaria risk algorithms vs performed mRDTs.

Statistical analysis	Original malaria risk assessment algorithms		ML malaria risk assessment algorithms	
—	(−) mRDT *n* = 160	(+) mRDT *n* = 320	(−) mRDT *n* = 160	(+) mRDT *n* = 320
Sensitivity, %	0.36	0.70	0.29	0.73
CI	0.30–0.41	0.63–0.76	0.15–0.44	0.61–0.84
Specificity, %	0.70	0.36	0.98	0.64
CI	0.63–0.76	0.30–0.41	0.95–1.0	0.52–0.76
PPV, %	0.63	0.43	0.87	0.67
CI	0.55–0.70	0.38–0.49	0.69–1.0	0.55–0.78
NPV, %	0.43	0.63	0.75	0.70
CI	0.38–0.49	0.55–0.70	0.66–0.83	0.58–0.82

Abbreviations: CI, Confidence Interval; ML, machine learning; mRDT, malaria rapid diagnostic test; PPV, positive predictive value; NPV, negative predictive value.

These data reveal that the newly generated THINKMD ML based malaria risk assessment algorithms overall improved point of care assessment of malaria risk compared to the original THINKMD malaria risk assessment algorithms used for the field-based study. Specifically, we were able to demonstrate that for falciparum malaria, even with only 280 “positive” patient encounters and 160 “negative” patient encounters, new ML algorithms can be generated and tested with prior study data that improve upon the current THINKMD malaria risk assessment algorithms. For positive mRDTs, ML significantly improved THINKMD malaria risk assessment specificity (36–64%) and PPV (43–67%) with a minimal increase in both sensitivity and NPV. For negative mRDTs, ML significantly improved THINKMD malaria risk assessments for specificity (70–98%), PPV (63–87%) and NPV (43–75%) with a decrease in sensitivity from (36–29%).

For diseases with non-specific symptomology, location as it pertained to temperature assessment could be identified as an important predictor during the development of new ML based malaria risk algorithms. Combining the location and symptom data resulted in the best performing random forest models as measured by out-of-sample F1 scores. The sensitivities and F1 scores increased consistently as more training data were added, indicating that the random forest models continue to benefit from “seeing” more patient encounters. THINKMD malaria risk algorithms had significantly higher specificity, but significantly lower sensitivity than the random forest model. Nevertheless, the resulting F1 scores were significantly higher for the random forest model.

Reviewing overall numbers of thermometer use in standard IMCI workflow consultations where temperature was recorded using a thermometer, 67 and 29% of children had positive and negative mRDT results, respectively, compared with 61 and 30%, respectively, in consultations with a history of fever but no temperature recorded. In THINKMD consultations with temperature taken with a thermometer, 38 and 16% of children had positive and negative mRDT results, respectively, and 44% had no mRDT performed. In THINKMD consultations with no thermometer readings, 64 and 33% of children had positive and negative mRDT results, respectively, and 2% had no mRDT performed.

To further assess the use of a thermometer on the assessment of malaria risk, data from a CHW with no thermometer was compared with data from a CHW with a thermometer. When using the standard workflow, 40% of consultations with a thermometer received an mRDT compared with 97% of consultations without a thermometer, Table 8.

**TABLE 8:** Comparison of THINKMD predictive statistics for a Community Health Worker (CHW) with dominant use and a CHW with low use of a thermometer.

	No thermometer assessment, *n* = 64	Thermometer assessments, *n* = 90
PPV	66%	50%
NPV	55%	91%
Sensitivity	88%	65%
Specificity	23%	53%
Total (+) mRDT	40 assessments, 62%	20 assessments, 15%
Total (−) mRDT	22 assessments, 34%	16 assessments, 10%
Total Not Administered	2 assessments, 3%	54 assessments, 75%

Abbreviations: CHW, community health worker; mRDT, malaria rapid diagnostic test; PPV, positive predictive value; NPV, negative predictive value.

THINKMD’s platform data showed that fever was associated with both malaria positive and negative mRDT results, and that vomiting, and headache were common in both groups.

## Discussion

There is currently a rapid evolution of digital mHealth platforms and their utilization for increasing health care capacity and access. The value of such platforms is that they can play a key role in accelerating scaling of primary healthcare delivery programs utilizing frontline community health workers while maintaining consistent quality.

Performing clinical risk assessment for acute febrile illnesses, specifically malaria, is important for population-based screening to determine patients at high risk, where diagnostic testing is indicated. In 2018, 412 million RDTS were sold globally (Organization, 2019). Therefore, the importance in obtaining these diagnostic tests and incorporating them into malaria risk assessments is critical for improving the delivery of appropriate treatment to those in need, while also decreasing the inappropriate utilization of other treatments such as antibiotics.

In this study, we were able to demonstrate that the utilization of embedded mRDT result acquisition resulted in a better understanding of the need for febrile-illness specific protocols, as well as consistent provision and use of necessary tools, like thermometers and RDTs, to rely more on factual evidence of disease, and less on self-reporting. This helps in two ways, first, to help FHW provide higher quality of care, and second, to improve algorithms to identify specific febrile illness using either manually modified or machine-learning algorithms based on measured symptomologies, geography, and other risks, more efficiently.

A potential key value of digital “physician-based” Bayesian integrated clinical risk assessment algorithms as developed and used in THINKMD’s mHealth platform is that they allow for continuous assessment of their accuracy when compared to field-based truth data. We were able to rapidly modify and test two different malaria specific logic approaches following initial use and analysis of assessment data that was confirmed with diagnostic mRDT testing, our current physician-based logic approach, and a new ML specific malaria logic using “prior” field-based data sets captured during this study. In each case, we were able to show significant improvements over the original THINKMD malaria assessment logic used for this study. This allows for continuous assessment and determination of required algorithm modifications as needed and allows for subsequent retesting using the same field-based data sets to assess if the modified algorithms have improved outcomes.

The importance of this approach is that these modifications can be improved quickly based on real field-data and tested immediately without the need for a new field-study. We were able to demonstrate this by making modifications to the original malaria risk algorithms into a modified malaria risk algorithm and testing and comparing the latter to the original algorithms using the same field-based clinical data sets and associated mRDT truth data sets. This provides the best approach to performing side by side algorithm modifications that can be rapid and avoid the delay and expense of a new field-based study to test the modified algorithms. IMCI and THINKMD malaria risk assessment algorithms both had strengths and weaknesses associated with their ability to identify children with and without malaria at an appropriate accuracy that would lead to capturing those children at high risk who should receive mRDT testing, and at a level that is both financially and logistically feasible for frontline healthcare delivery programs.

The development of ML based clinical risk assessment algorithms has significant potential with respect to discerning different disease risk for acute febrile illnesses where clinical data elements have a high degree of overlap, and diagnostic testing for all the possible etiologies is expensive and not universally available. It is critical to utilize an approach that can incorporate supervised ML statistical learning methods that have as its foundation proven clinical validated algorithms that can utilize field-based clinical data, assessment data and field-truth confirmatory diagnostic data. The current THINKMD integrated clinical assessment algorithms were designed to be modified as described above as well as to be utilized for developing new ML based clinical assessment algorithms. In this study, we were able to develop new supervised ML malaria risk assessment algorithms using this approach that in many respects significantly improved the quality of the clinical assessments. The additional value of this approach is that each assessment can be analyzed specifically on how each was generated and how each clinical data element contributed. This allows for specific clinical review of the new ML based algorithms and how they differ from the ones that were used for training. Of importance for this study was that this was accomplished using a small data set of 555 clinical assessments and 480 administered mRDTs.

One major challenge in the approach to febrile illness is that the presentation of fever, whether self-reported or measured, is expected to result in antimicrobial prescription, by both health workers and caregivers, even if it is not appropriate. As IMCI is not clear in identifying those who do not need antibiotics, health workers are deferring to provide antibiotics independent of mRDT results. In one study, artemisinin-based combination therapies (ACTs) prescribed singly or in combination with an antibiotic occurred in 98.7, 18.5 and 25.8% of patients with a positive test, negative test and those not tested, respectively (Unitaid, 2018). The improvement of frontline acute febrile risk assessments, such as malaria, have the potential to reduce antimicrobial resistance through more accurate diagnosis and appropriate antimicrobial stewardship. Consistent with WHO recommendation, in 2011, Nigeria updated the National Malaria Treatment Guidelines to reflect WHO’s test-and-treat policy for malaria. While this resulted in more targeted use of ACTs inadvertently, in many countries an overuse of antibiotics could be observed in malaria negative patients (Hopkins et al., 2017).

The current work suggests that even during access shortages to mRDTs, using the improved malaria risk assessment algorithm, health workers will be able to complete a more accurate assessment and provide more appropriate treatment.

## Limitations

There were confounding variables to the analysis of malaria risk assessment and mRDT analysis that may influence the results. First, not all CHW were equipped with thermometers (*n* = 2 of 7). As fever was the driving data point for IMCI malaria-risk, this fact had great influence on PPV/NPV of both IMCI and THINKMD malaria risk assessments and subsequent mRDT administration. Secondly, IMCI thresholds for “malaria risk” were used in the THINKMD mHealth platform to trigger the mRDT panels and determine if an mRDT would be administered (history of fever, axillary temperature above 37.5°C); children who were deemed by IMCI guidelines to not have a “risk of malaria” did not receive an mRDT to confirm this assessment. Additionally, as with all diagnostic tests, mRDTs have false positives and false negatives. Given the increased reliance on mRDTs for making clinical decisions, we chose to use them as the ground truth for evaluating the various diagnostic approaches considered in our analysis. However, future work should focus on the agreement (or lack thereof) between mRDT, symptom-based assessment, PCR, and blood-smear-microscopy-based diagnoses.

Of note is that our current malaria logic was tested in predominately low risk regions with physicians as the gold standard in 1,000 patient encounter in five different countries and correlated 90% of the time with a local physician’s assessment (Finette et al., 2019). The data shown above provides evidence that physician/frontline health professionals and worker assessments for malaria, without the support of mRDTs has significant limitations. For this study, we cannot conclude that if physicians were assessing malaria and using mRDTs that they would correlate with THINKMD’s malaria risk assessment, as this portion of the study was not performed. As a result of the malaria risk assessment and mRDT data acquired in this study, THINKMD can review these findings and make modifications to its malaria specific logic in order to improve all the key clinical assessment parameters, based on the Who’s 2010 high and low malaria risk guidelines stating the need to “improve the rational use of ACTs, all suspected cases of malaria should have a parasitological confirmation before treatment.

## Conclusion

This study demonstrates that integrated clinical digital algorithms such as those used in the THINKMD mHealth platform can be modified and quickly tested using previously acquired field-based clinical data elements and diagnostic truth data such as mRDTs. The ability to rapidly develop better mHealth logic, without requiring new large-scale field-based study testing is novel and could prove extremely valuable for developing high quality frontline point-of-care clinical assessment platform for complex clinical conditions and disease. This approach can also be used to develop new supervised ML based clinical risk assessment algorithms as well, which in the long-term will likely prove to be much more valuable in performing population risk assessments for complex diseases such as respiratory distress/pneumonia, sepsis and differentiating risk potential for a wide variety of acute febrile illnesses.

## Data Availability

The raw data supporting the conclusion of this article will be made available by the authors, without undue reservation.
