# Supplementary material for: Development and Validation of Manually Modified and Supervised Machine Learning Clinical Assessment Algorithms for Malaria in Nigerian Children
Source: Front Artif Intell. 2022 Feb 3;4:554017. doi: 10.3389/frai.2021.554017 (PMC8851346; doi:10.3389/frai.2021.554017)
Supplement: Supplementary file 1 [file DataSheet1.PDF]

Input Variables

Variable	Type
muac green	Yes/No
head bobbing no	Yes/No
nasal flaring or retractions no	Yes/No
skin infection none	Yes/No
sleeping less	Yes/No
swelling of the feet no	Yes/No
why is the child being seen fever	Yes/No
pale eyelids no	Yes/No
eha code code	Yes/No
diarrhea none	Yes/No
vomiting severity none	Yes/No
rash no	Yes/No
tears unknown	Yes/No
geo location lat	Latitude
geo location long	Longitude
wheezing no	Yes/No
red eyes no	Yes/No
nasal discharge yes	Yes/No
breathing normal	Yes/No
coughing none	Yes/No
geo permission message	Yes/No
eha site kantudu hp	Yes/No
skin turgor normal	Yes/No
disposition calm	Yes/No
drinking normal	Yes/No
chest indrawing no	Yes/No
pain when moving neck no	Yes/No
language en US	Yes/No
gender male	Yes/No
capillary refill normal	Yes/No
urinating normal	Yes/No
disclaimer checked	Yes/No
sleeping normal	Yes/No
why is the child being seen seizure or fit	Yes/No
why is the child being seen headache	Yes/No

tears yes	Yes/No
wheezing yes	Yes/No
breathing more	Yes/No
coughing mild	Yes/No
eha site unguwa jakada hp	Yes/No
gender female	Yes/No
muac red	Yes/No
diarrhea mild	Yes/No
vomiting severity mild	Yes/No
tears no	Yes/No
eha site hotoro danmarke	Yes/No
latching normal	Yes/No
rash yes	Yes/No
nasal discharge no	Yes/No
why is the child being seen foul smelling urine	Yes/No
pale eyelids yes	Yes/No
red eyes yes	Yes/No
muac yellow	Yes/No
why is the child being seen cold chills	Yes/No
sleeping more	Yes/No
why is the child being seen bloody stools	Yes/No
drinking less	Yes/No
why is the child being seen sick	Yes/No
drinking much less	Yes/No
chest indrawing yes	Yes/No
why is the child being seen pain when urinating	Yes/No
eha site tudun murtala hc	Yes/No
swelling of the feet yes	Yes/No
latching less	Yes/No
why is the child being seen aching	Yes/No
urinating less	Yes/No
urinating unknown	Yes/No
why is the child being seen ear pain	Yes/No
muac orange	Yes/No
skin turgor less	Yes/No
capillary refill less	Yes/No
head bobbing yes	Yes/No

pain when moving neck yes	Yes/No
disposition irritable	Yes/No
nasal flaring or retractions yes	Yes/No
wheezing unknown	Yes/No
wakefulness none	Yes/No
disposition sleeping	Yes/No
eha site alfindiki hc	Yes/No
drinking more	Yes/No
vomiting severity moderate	Yes/No
why is the child being seen none	Yes/No
diarrhea moderate	Yes/No
breathing much more	Yes/No
coughing moderate	Yes/No
urinating much less	Yes/No
muac unknown	Yes/No
diarrhea	Yes/No
vomiting severity	Yes/No
wakefulness moderate	Yes/No
capillary refill much less	Yes/No
eha site dala mch	Yes/No
skin infection warm	Yes/No
skin infection red	Yes/No
pale eyelids unknown	Yes/No
skin infection swelling	Yes/No
skin infection pain	Yes/No
skin turgor much less	Yes/No
latching much less	Yes/No
geo location error	Yes/No
nasal flaring or retractions unknown	Yes/No
chest indrawing unknown	Yes/No
head bobbing unknown	Yes/No
skin infection discharge	Yes/No
swelling of the feet unknown	Yes/No
rash unknown	Yes/No
wakefulness	Yes/No
red eyes unknown	Yes/No
nasal discharge unknown	Yes/No
pain when moving neck unknown	Yes/No
language en US 1	Yes/No

weight kg	Numeric
respiratory rate ile	Numeric
weight ile	Numeric
heart rate ile	Numeric
respiratory rate bpm	Numeric
age days	Numeric
heart rate bpm	Numeric
